# Inkjet-Printed LSM-YSZ Thin Films for Enhanced Oxygen Electrodes in Solid Oxide Fuel Cells

**DOI:** 10.1021/acs.energyfuels.4c00673

**Published:** 2024-07-12

**Authors:** Michalis Charalampakis, Leila Zouridi, Ioannis Garagounis, Anastasios Vourros, George E. Marnellos, Vassilios Binas

**Affiliations:** † Department of Chemistry, University of Crete, Vasilika Vouton, Heraklion 70013, Greece; ‡ Institute of Electronic Structure and Laser, Foundation for Research and Technology-Hellas, N. Plastira 100, Vasilika Vouton, Heraklion, Crete 70013, Greece; § Department of Materials Science and Technology, University of Crete, Vasilika Vouton, Heraklion 70013, Greece; ∥ Chemical Process & Energy Resources Institute, Centre for Research & Technology Hellas, sixth km Harilaou-Thermis, Thessaloniki 57001, Greece; ⊥ Department of Chemical Engineering, Aristotle University of Thessaloniki, University Campus, Thessaloniki 54124, Greece; # Physical Chemistry Laboratory, Chemistry Department, Aristotle University of Thessaloniki, Faculty of Sciences, Thessaloniki 54124, Greece

## Abstract

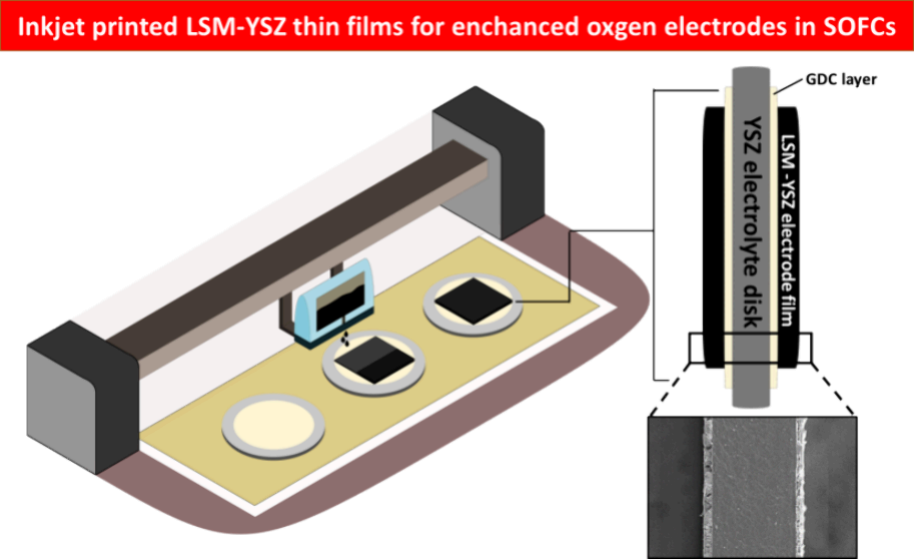

In the present work, symmetrical oxide ion conducting solid oxide single cells with inkjet-printed composite LSM-YSZ electrodes, onto commercially available YSZ dense substrates using GDC as buffer interlayer, were fabricated and characterized. Stable inkjet-printable LSM-YSZ nanoparticle inks were developed based on water solvent, after processing with high intensity ball milling. The deposition of LSM-YSZ electrodes was performed by inkjet printing, as well as a conventional additive manufacturing technique, screen printing, in order to compare the electrochemical performance of the produced cells for the reversible charge transfer reaction (O_2_ + 4 e^–^ ↔ 2 O^2–^). The physicochemical properties of the LSM-YSZ nanoparticle ink was investigated to determine ink printability. The electrochemical performance of fabricated inkjet-printed and screen printed symmetrical cells (LSM-YSZ | GDC | YSZ | GDC | LSM-YSZ) exposed under a synthetic air atmosphere was evaluated in a single chamber cell reactor, employing the AC impedance spectroscopy and linear scan voltammetry techniques, at the temperature range of 700–850 °C. The inkjet-printed electrodes exhibited highly homogeneous and porous morphologies with the corresponding cell achieving current densities almost five times higher, up to 1 A/cm^2^ at 2 V cell potential and 850 °C, than those of the equivalent screen-printed one. To the best of our knowledge, this is the first successful implementation of water-based inks of LSM-YSZ electrodes in the fabrication of inkjet-printed solid oxide cells.

## 1 Introduction

A solid oxide fuel cell (SOFC) constitutes a promising electrochemical technology to directly convert the chemical energy contained in fuels into electrical energy, bypassing the Carnot thermodynamic limitations governing the classical thermal engines. Due to their high energy conversion efficiency, and thus low environmental footprint per produced power unit, SOFC technology has attracted much focus in the energy research community.^1^ Moreover, SOFCs are fuel flexible since they operate at high temperatures where, apart from pure hydrogen, a wide variety of gaseous (e.g., natural gas, syngas, biogas, etc.), liquid (gasoline, diesel, bioethanol, synthetic liquid fuels, etc.), and solid fossil fuels or biofuels (e.g., coal, primary biomass, etc.) can be utilized as feedstock with some modifications on the integrated SOFC system (e.g., purification, evaporation, feeding system, etc.).^1,2^ These advantageous characteristics have rendered SOFCs as the next generation energy conversion technology toward a more sustainable future.

The necessity to employ thin films for the fabrication of the different cell counterparts in SOFCs (i.e., electrolyte, electrodes, interconnects) has been well acknowledged in the literature, in order to sufficiently lower their operation temperature, while maintaining high ionic and electronic conductivities, thus enhancing efficiency.^3^ Operation at lower temperatures could lead to the employment of more available and cost-effective materials in SOFCs, especially for the device casing and electronic contacts, as well as widen the spectrum of their applications.^4−6^ Fabrication techniques employed in publications reporting on thin film SOFCs involve the use of plasma (thermal) spraying, pulsed laser deposition (PLD), electrophoretic deposition (EPD), chemical vapor deposition (CVD), and magnetron sputtering, with results of great scientific and technological importance.^7−9^ However, the application of the aforementioned methods at larger scales is still facing major challenges due to the inherent difficulties imposed by their process complexity. In addition, fabrication costs, environmental footprint, repeatability, and fidelity of products are challenging aspects that need to be considered, when searching for a suitable and effective ceramics processing technique. Moreover, methods that are flexible in their implementation at different manufacturing steps, are preferable for in-line continuous fabrication sites.

Additive manufacturing (AM) techniques have been proposed as a sufficient solution for both fast prototyping and scalable manufacturing of thin film devices.^10,11^ Their main advantages lie in the contact-less nature of layer-by-layer deposition, as well as fabrication automation, reduction of waste products, and high configurability to optimize deposition precision.^12^ In the case of two-dimensional thin film deposition, from organic to ceramic materials, inkjet printing (IJP) has gained a lot of scientific interest in the past decade, due to its facile, cost-effective, and flexible features.^13−16^ In the field of SOFCs, IJP has been utilized by researchers in a limited number of works so far,^17^ with very promising results.^18−20^ Since the implementation of IJP in SOFC fabrication is state of the art, the vast majority of reports refer to partially printed SOFC single cells,^21−25^ either the electrolyte or electrode counterparts, with rare exceptions of fully inkjet-printed devices.^26−28^

The most common cathode materials as oxygen electrodes in SOFCs are perovskite-type mixed oxides, such as Sr-doped LaMnO_3_ (LSM), which is an intrinsic p-type conductor. Doping of LaMnO_3_ with Sr^2+^ is necessary, since the doped material exhibits a matching thermal expansion to that of the yttria-stabilized zirconia (YSZ) electrolyte, as well as increased mixed ionic and electronic conductivity, with a surface-mediated oxygen transport mechanism governing at lower overpotentials.^29^ In order to further enhance the ionic conductivity of the electrode and extend the active electrochemical zone for the oxygen reduction reaction (ORR) at the cathode, the so-called three-phase boundary (TPB), an electrolyte material such as the conventional YSZ, is mixed with LSM, forming a composite cathode electrode. LSM-YSZ exhibits sufficient electronic and oxygen ion conductivity, making it a cathode material of utmost interest in SOFCs.^30^ Due to these properties, LSM-YSZ is currently the standard cathode material for SOFCs operating at temperatures above 700 °C.

Since IJP is easily compatible with many different materials, there are several reports using a variety of cathode composites such as LSM-YSZ,^20,27,28^ LSCF-GDC,^23,31^ PBSCF,^26^ and SSC-SDC,^32^ although there are few relevant literature studies overall. For LSM-YSZ specifically, a work by Farandos et al. reported peak power density (PPD) values up to 690 mW·cm^–2^ at 788 °C, with inkjet-printed LSM-YSZ electrode layers of 150 μm thickness, utilizing a butanol-based inkjet ink.^20^ However, when the same group switched from organic- to water-based inks, their deposited films failed due to delamination even at thermal treatment temperatures below 550 °C. Another report utilizing a water-based inkjet ink to fabricate LSM-YSZ electrode layers is the conference proceeding by Da’as et al., who used IJP to infiltrate YSZ porous layers with an LSM precursor solution, with unfortunately no electrochemical data being reported.^33^ To the best of our knowledge, no works have been reported so far on a successful implementation of LSM-YSZ water-based inks to deposit thin films as cathode electrodes for SOFCs.

In the present study, we report on the deposition of high-performance and high-quality LSM-YSZ thin films as cathode layers for SOFCs by utilizing IJP technology, based on the various advantages of the IJP method described above. Symmetrical LSM-YSZ single cells for SOFCs were successfully fabricated by IJP on commercial YSZ electrolyte substrates with a screen-printed GDC buffer interlayer. LSM-YSZ nanoparticles were produced by processing of a commercial powder via high-intensity ball milling. Subsequently, a water-based ink containing LSM-YSZ nanoparticles was formulated and its physicochemical properties of viscosity, density, surface tension, and particle distribution were examined, in order to verify its printability by inkjet printing and stability over time. For the film depositions, an IJP system with a piezoelectric drop-on-demand (DoD) print-head was utilized. Parameters of the print-head controlling the deposition such as operating temperature, jetting voltage, and the jetting waveform were optimized to achieve the continuous generation and uniformity of droplets toward stable inkjet-printing. The use of a single jetting nozzle was established to optimize the printing process, resulting in depositions with better accuracy. For the screen-printed electrodes, a conventional terpineol-based mixture of LSM-YSZ was utilized and symmetrical cells were fabricated. Finally, the electrochemical performance of symmetrically inkjet-printed (IJP) LSM-YSZ|GDC|YSZ single cells was evaluated under a synthetic air atmosphere at a temperature range of 700–850 °C and compared to the equivalent screen-printed (SP) cells. In all examined temperatures, the inkjet-printed cell showed substantially better electrochemical performance compared to the screen-printed cell.

## 2 Experimental section

### 2.1 Materials

The cathode material used for the electrode films was a 50/50 wt % composite of lanthanum strontium manganese (La_0.2_Sr_0.8_MnO_3_, LSM)–yttria-stabilized zirconia (8 mol % YSZ) oxide powder (Fuel Cell Materials, USA). The printing substrates, also from Fuel Cell Materials, were solid electrolyte 8 mol % YSZ disks with a 2 cm diameter and 240 μm thickness. For the formulation of the water-based nanoparticle ink, propylene glycol (C_3_H_8_O_2_) and Triton X-100 (C_14_H_22_O(C_2_H_4_O)_*n*_), both from Merck, were used. A GDC (Gd_0.2_Ce_0.8_O_3_) powder and a commercial terpineol-based ink vehicle (both from Fuel Cell Materials, USA) were used for the deposition of the GDC interlayers and LSM-YSZ electrodes in SP cells.

### 2.2 Ball Milling Processing of Powders

An aqueous LSM-YSZ dispersion was prepared by high-intensity ball milling in a zirconia vessel (85 mL volume capacity) with zirconia beads (0.5 mm diameter) as the grinding medium (Planetary Micro Mill, Pulverisette 7, Fritsch). This processing was employed to reduce and homogenize the average particle diameter and obtain a dense aqueous dispersion. Typically, 3 g of LSM-YSZ powder were mixed with 25 mL deionized water and 100 g of the grinding media and then milled for 30 min at 700 rpm, resulting in a dense LSM-YSZ nanoparticle dispersion, subsequently filtered through a syringe filter (0.45 μm nylon membrane).

### 2.3 Preparation and Physicochemical Characterization of Water-Based Inkjet ink

For the formulation of the LSM-YSZ nanoparticle ink, the as prepared dense aqueous dispersion was mixed with propylene glycol at a volume ratio of 6:4 and then 0.5 mg/mL of Triton X-100 was added dropwise. The pH value was adjusted by the dropwise addition of dense HCl acidic solution, as the print-head of the inkjet printer is prone to corrosion for pH values greater than 9. In addition, higher ionic concentration contributes to lower reagglomeration rates, thus increasing the ink stability. Finally, the mixture was stirred until a homogeneous dark gray ink was obtained. The hydrodynamic particle diameter was examined by dynamic light scattering on a Malvern Zetasizer Nano ZS90 by preparing samples with 15 μL of the nanoparticle ink (at different days of storage) diluted in 2 mL of water solvent for scattering measurement at 90° to be accurate. Thermogravimetric analysis was performed in a TGA SDT Q600 V8.3 Build 101, in Argon flow (100 mL/min), with a 5 °C/min heating ramp. The goniometer used for contact angle (sessile drop) and surface tension (pendant drop) measurements was the OCA35 by Dataphysics. Viscometry was conducted using a DMA 4100 M density meter from Anton Paar with a Lovis 2000 ME microviscometer extension and a 0.59 mm stainless steel ball inside the capillary, at six different temperatures (25–50 °C, by increments of 5 °C).

### 2.4 Fabrication by Screen Printing of LSM-YSZ Electrodes and GDC Interlayers

For the purpose of comparison with the IJP technique, symmetrical cells with screen-printed LSM-YSZ electrodes were also developed. For the case of the barrier interlayer between the electrolyte substrate and the LSM-YSZ electrode film, GDC powder was mixed with a terpineol-based ink vehicle in a mortar at a 70:30 wt % ratio, and a single layer of 1.8 cm^2^ apparent surface area was screen printed on each side of the YSZ disks, followed by sintering at 1350 °C for 3 h with a 2 °C/min heating rate. For the preparation of the LSM-YSZ screen-printed electrode films, the ink was prepared by mixing LSM-YSZ powder with the same ink vehicle used for the GDC interlayer. The LSM-YSZ powder to ink vehicle weight ratio was 60:40 wt %, and two layers per side were deposited on top of the presintered interlayers, with a drying step of 30 min at 90 °C between the first and the second deposited layer. The sintering procedure was the same as that for the IJP samples (see section below).

### 2.5 Fabrication by Inkjet Printing of LSM-YSZ Electrodes

A Dimatix DMP-2850 drop-on-demand (DoD) material printer by Fujifilm, equipped with a 10 pL piezoelectric cartridge (DMC-11610), was used to print LSM-YSZ patterns. For the fabrication of the symmetric inkjet-printed electrodes on the commercial YSZ substrates, initially, a 1.2 μm layer of GDC was deposited on both sides of the electrolyte disks by screen printing (see section above). The formulated LSM-YSZ nanoparticle ink was used to print squared thin films of 1 cm^2^ area, by printing 90 layers symmetrically on both sides of the GDC|YSZ|GSC disks. Printing was conducted at optimized parameters: cartridge temperature of 40 °C, jetting voltage of 30 V, substrate temperature of 60 °C, printing height of 300 μm, interlayer delay equal to 8 min, and drop spacing of 31 μm. Finally, the symmetric cells were sintered in air at 1100 °C for 2 h (heating rate 2 °C/min).

### 2.6 Electrochemical Measurements

The electrochemical performance of both inkjet-printed (IJP) and screen-printed (SP) LSM-YSZ symmetrical cells was assessed in a homemade single chamber reactor cell (Figure S5), employing AC impedance spectroscopy and linear scan voltammetry measurements. The custom-made single chamber reactor cell consists of a stainless-steel head, which incorporates specific provisions for the inlet and outlet flows, to which a quartz tube (120 mm long, ID = 28 mm, OD = 30 mm) is accommodated. The quartz tube is equipped with a cooling ring (SS 316) and a Viton O-ring for sealing. In each experiment, the cell was located inside the quartz tube, and both electrodes were exposed to the same synthetic air atmosphere. Two thin Au wires contacting the electrodes were used to hold the cell suspended inside the quartz tube and to electrically connect the cell to the Versa Stat 4 electrochemical workstation. Quartz tubes (ID = 1 mm, OD = 3 mm) were employed to insulate the Au wires from the reactor head and Ultra-Torr fittings held the tubes in place. The top of these tubes was sealed by placing and melting at open flame a small (about 1 cm long) piece of 1/8″ polyethylene tube. Electrode polarization curves (scan rate 25 mV/s) and AC impedance spectra (frequency: 600 kHz–100 mHz, amplitude: 30 mV RMS) were obtained employing a Versa Stat 4 electrochemical workstation by Princeton Applied Research and the relevant software (Versa Studio) for data processing. All electrochemical measurements were conducted under atmospheric pressure, at temperatures between 700 and 850 °C and the feed flow rate of synthetic air (Air Liquide) equal to 150 cm^3^/min.

### 2.7 Powder and Thin Film Physicochemical Characterization Techniques

X-ray diffraction (XRD) was conducted on a Bruker D8 Advance copper anode diffractometer (CuKa radiation) equipped with a nickel foil monochromator. X-Pert Highscore Plus was used to analyze and identify the diffraction patterns. Scanning electron microscopy (SEM) was conducted on a JEOL 7000 electronic microscope operated at 20 kV.

## 3 Structural and Morphological Characterization of LSM-YSZ Nanoparticles

**Figure 1:**
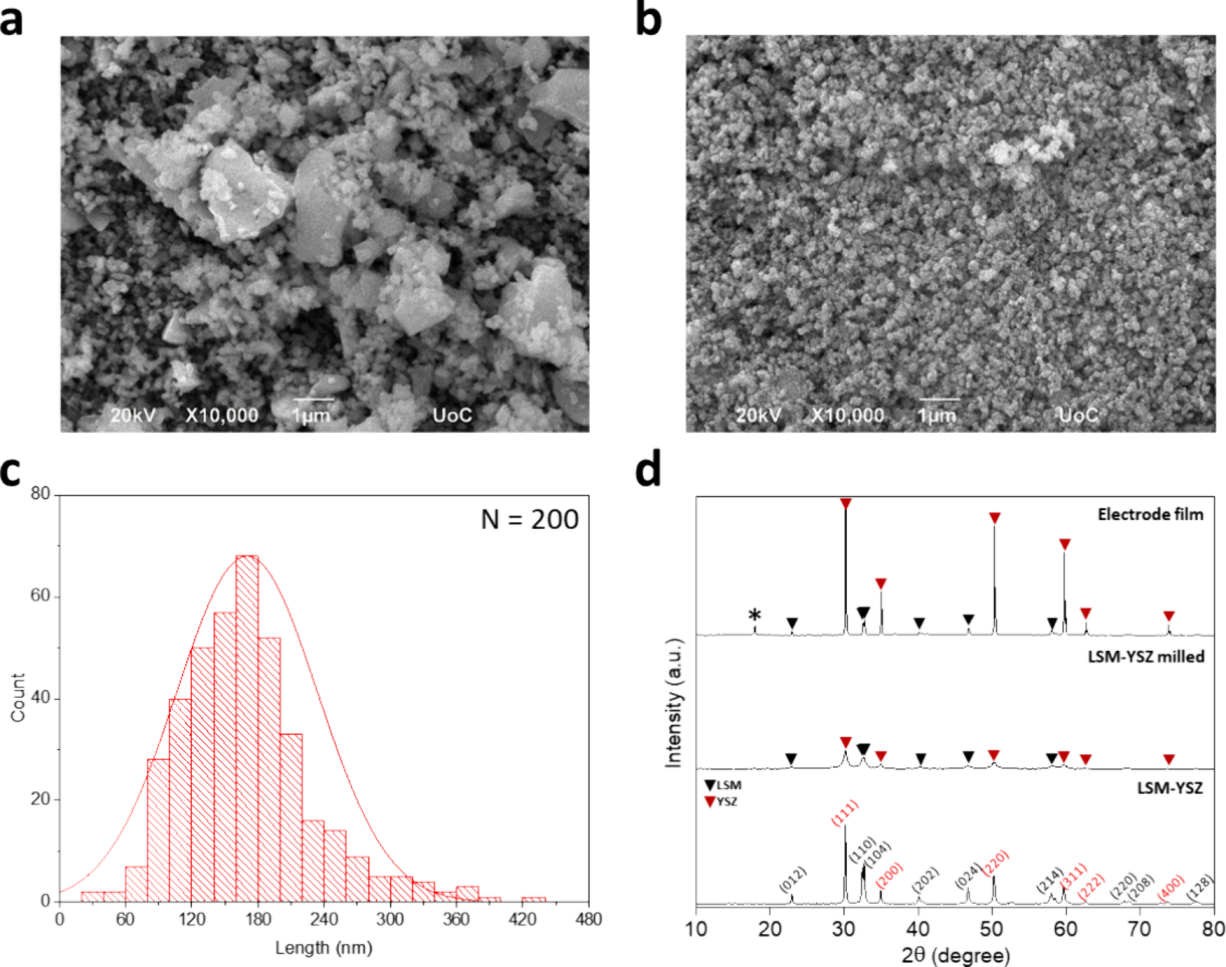
SEM images of LSM-YSZ powder before (a) and after ball milling (b), particle size distribution after ball milling process, (c) and X-ray diffraction patterns (d).

The most crucial step in the development of the IJP process is the formulation of stable and printable inks. To achieve optimum printing conditions and minimize nozzle blockage-related challenges, particle sizes below 1/50th of the nozzle diameter are recommended. The employed print-head cartridge is equipped with 21.5 μm nozzles, which suggests that the average particle diameter should not exceed 430 nm. Figure 1 shows the morphological and structural characteristics of the commercial LSM-YSZ powder and LSM-YSZ nanoparticles produced after the ball milling process. LSM-YSZ powder before ball milling is polydispersed with particle sizes that vary greatly from 100 nm to 3 μm (Figure 1a). On the contrary, the produced LSM-YSZ milled powder (Figure 1b) exhibits a greatly reduced, much narrower particle size distribution of 100–200 nm with an average diameter of 170 nm (Figure 1c). Moreover, EDX stoichiometric analysis confirms a similar elemental ratio of the produced LSM-YSZ powder to the parental material, confirming that the grinding process did not alter the chemical composition of the pristine powder (Figure S1). In addition, X-ray diffraction was conducted to verify the structure and crystallinity of the LSM-YSZ powder before and after ball milling. As seen in Figure 1d, the characteristic 2-theta peaks at 22.9, 32.4, 32.7, 40, 46.7, 58, 67.9, 68.5, and 77.8° correspond to crystal planes (012), (110), (104), (202), (024), (214), (220), (208), and (128) of rhombohedral LSM perovskite mixed oxide, while 2-theta peaks at 30.1, 34.9, 50.1, 59.6, 62.5, and 73.7° correspond to the (111), (200), (220), (311), (222), and (400) planes of cubic YSZ, respectively. The produced nanoparticles are stable after the process despite the significant reduction in crystallinity as indicated by the lower intensity and broadening of the diffraction peaks, a behavior that is ascribed to the decrease of particle size to the nanorange. However, high crystallinity is restored after annealing the films at high temperatures.

## 4 Physicochemical Properties of LSM-YSZ Nanoparticle Inks

For the formulation of the water-based ink, certain physicochemical properties inherent to water, in particular viscosity and surface tension, have to be fine-tuned. Despite being a low-cost, nontoxic, universal solvent, water is not suitable for inkjet printing without the use of regulating additives. In the present work, propylene glycol was used as a viscosity modifier and particle dispersant, while triton X-100 (a nonionic surfactant) and hydrochloric acid were added in order to adjust surface tension and pH, respectively. In addition to IJP manufacturer recommendations, a well-established method in the literature for evaluating the printability of any ink or suspension^13,17^ is the calculation of the dimensionless number *Z*, i.e., the inverse of the Ohnesorge number, defined as
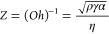
1where ρ is the density, γ the surface tension, μ the viscosity, and α is the characteristic length scale (in this case the nozzle diameter).^34,35^ This number correlates the inertial forces, surface tension, and viscous forces for drop formation in DoD systems and ideally ranges between 1 and 10.^36^

**Figure 2:**
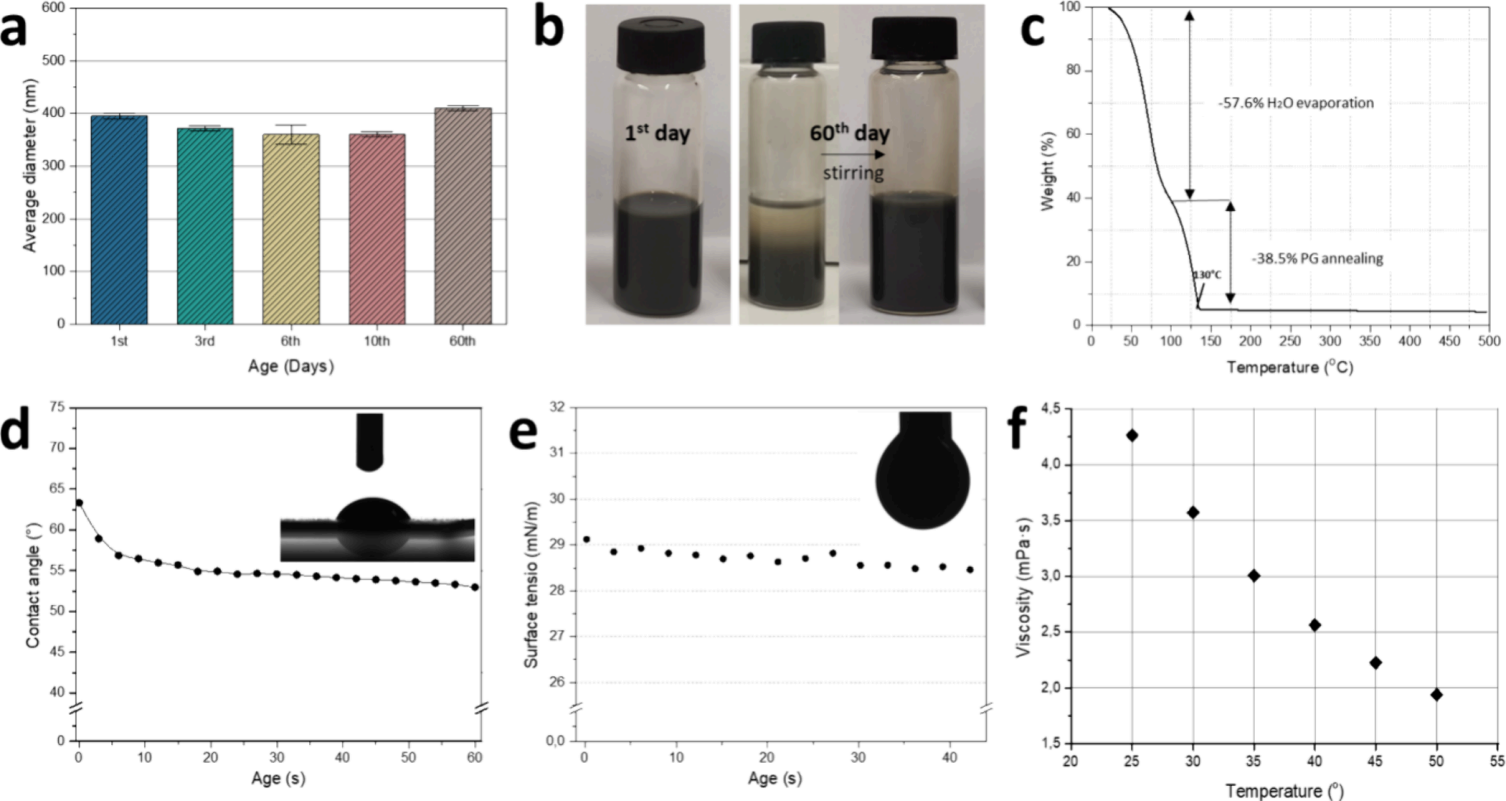
(a) Average hydrodynamic nanoparticle diameter over time, (b) LSM-YSZ ink on the 1st and 60th day of storage, (c) thermogravimetric curve with annotations of weight loss, (d) contact angle of sessile drop on GDC|YSZ substrate, (e) surface tension of pendant drop, and (f) ink viscosity in relation to temperature.

The hydrodynamic diameter of the suspended LSM-YSZ nanoparticles was measured during the first 10 days after ink formulation, as well as 60 days of storage, to assess the long-term stability of the produced ink. As seen in Figure 2a, the formulated LSM-YSZ ink exhibits excellent stability with an average hydrodynamic diameter of about 370 nm, lower than the aforementioned recommended limit. The average diameter remains similar even after 2 months of storage, with no aggregation of nanoparticles being observed owing to the ink’s excellent stability over time. While partial precipitation is observed over this period, the suspension of nanoparticles can be restored by a brief stirring step (Figure 2b). Thermogravimetric analysis reveals two stages of ink decomposition, as indicated by the two consecutive sharp weight loss steps in Figure 2c. At first, on temperatures up to 100 °C, a 57.6% weight loss due to water evaporation takes place. Then, at temperatures between 100 and 130 °C, another weight loss of 38.5% takes place, which is attributed to the decomposition of propylene glycol. Finally, above 130 °C, LSM-YSZ powder accounts for the remaining 3.9 wt %, from which a 46.03 mg/mL nanoparticle ink concentration can be deduced. Since the volumetric amount of Triton X-100 used during the preparation of the ink was insignificant compared to the other employed solvents, there is no visible weight loss curve associated with its decomposition (boiling point at 270 °C).

In Figure 2d the wettability of the formulated LSM-YSZ ink on the GDC|YSZ substrate is examined. Drop spreading and its good affinity with the substrate are vital for successful deposition with IJP. In this case, the average contact angle ranges around 55°, which is regarded as a good value for IJP deposition. The surface tension of ink droplets was also measured at 28.71 mN/m (Figure 2e), much lower than that of water (72 mN/m). Finally, to assess the printability and rheological characteristics of the ink, viscosity measurements were performed at different temperatures, as depicted in Figure 2f. A near linear decrease of viscosity in regards to temperature, ranging from 4.25 mPa·s at 25 °C down to 1.9 mPa·s at 50 °C, is observed.

Table 1 summarizes the rheological properties of the formulated LSM-YSZ water-based ink at different temperatures. The inverse Ohnesorge number *Z*, is also calculated and falls within the desired printable range, for temperatures ranging from 25 to 40 °C. As the temperature increases, *Z* exceeds the value of 10, where large column extensions and satellite droplets are formed, downgrading the printing quality. This is mainly due to the reduction in viscosity.^37^ For the LSM-YSZ ink, extensive tests were carried out on droplet formation and jetting in order to produce stable, consistent drops, which are essential for good printing quality and repeatability. A range of operating temperatures were tested in conjunction with other crucial parameters, such as the jetting voltage and jetting waveform with the best results being achieved at 40 °C.

**Table 1:** Physichochemical Properties and *Z* Number of the Water-Based LSM-YSZ Nanoparticle Ink for Different Operating Temperatures

T [°C]	ρ [kg m^–3^]	γ [10^–3^ N m^–1^]	η [10^–3^ Pa s]	α [10^–6^ m]	*Z*
25	1072.8	28.71	4.3	21.5	6.03
30	1069.7		3.6		7.19
35	1066.1		3.0		8.53
40	1061.8		2.6		9.98
45	1056.8		2.2		11.47
50	1051.4		1.9		13.15

## 5 Inkjet Printing of LSM-YSZ Thin Films

**Figure 3:**
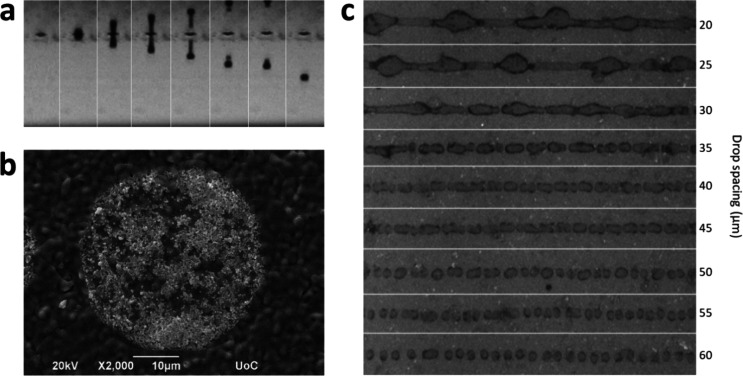
(a) Drop formation as seen by the drop-observation camera of the inkjet printer, (b) SEM image of a single deposited drop, and (c) images of LSM-YSZ lines printed with different drop spacings.

As seen from optical images captured using the drop-observation camera of the inkjet printer (Figure 3a), the LSM-YSZ nanoparticle ink exhibits ideal drop formation at optimized jetting parameters. The drops are perfectly round and symmetrical with the initial formation of a small drop extension (tail) that subsequently merges with the main droplet body, without forming any satellite droplets. The resulting deposition of such a drop on the substrate is shown in Figure 3b, as observed by SEM, and its diameter is measured at 39 μm. Images of LSM-YSZ lines printed on GDC|YSZ substrates with different drop spacing values (i.e., the distance between consecutive drops, which defines print resolution) are presented in Figure 3c. The most uniform and coherent lines are formed when using a drop spacing about 30 μm, which is roughly equivalent to a droplet overlap of consecutive drops by 23%, as defined by Kwon et al.^38^ At lower drop spacing values, lines are uneven with big bulges forming due to significant overlap of consecutive drops (e.g., 36% overlap at 25 μm drop spacing), while at higher values, the printed lines are discontinuous since deposited drops are too distant to merge (e.g., −2.6% overlap at 40 μm drop spacing). Based on the SEM images of printed lines at different drop spacing values (Figure S2), the pattern resolution was set at 819 DPI (i.e., 31 μm drop spacing).

**Figure 4:**
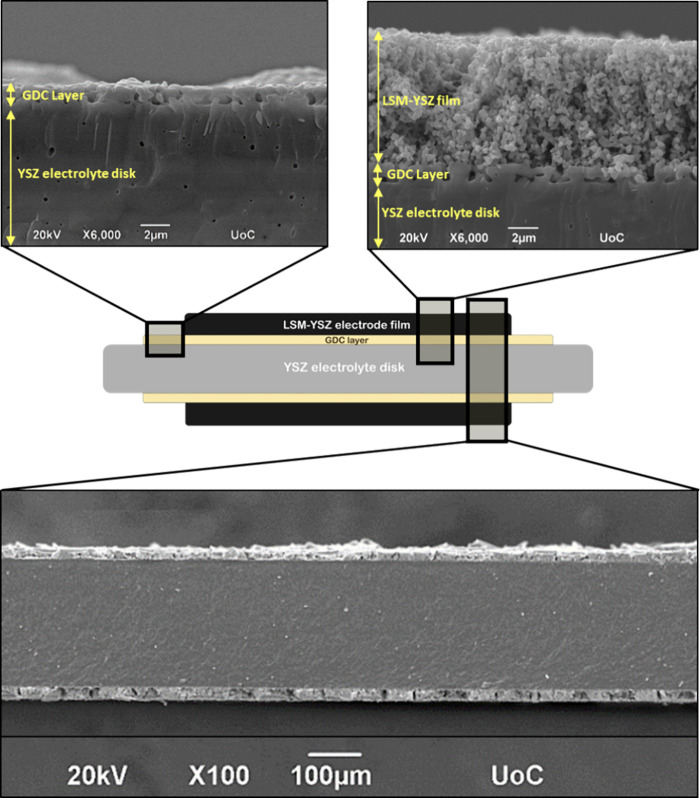
Schematic illustration of a LSM-YSZ symmetrical cell and cross-sectional views of the different layers and interfaces (9 μm thickness of IJP LSM-YSZ electrode film).

A schematic illustration of the IJP symmetrical cell cross-sectional view of the different layers and interfaces as observed by SEM is presented in Figure 4. As seen in the respective SEM images, both the GDC layer and LSM-YSZ electrode film show excellent adhesion to the YSZ substrate as well as uniformity.

**Figure 5:**
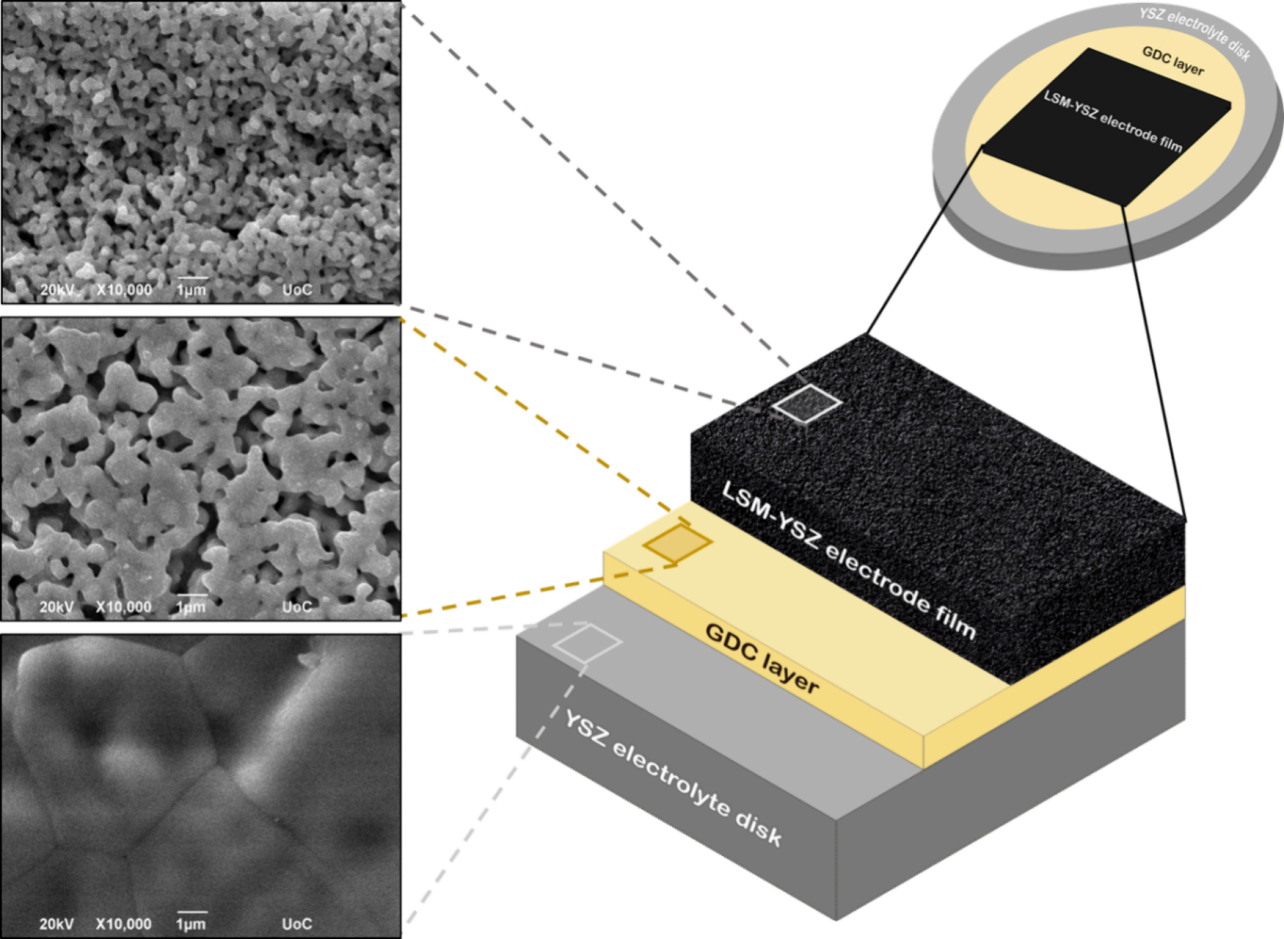
Schematic illustration and top view SEM images of the different layers of a half-cell with screen-printed GDC interlayer and inkjet-printed LSM-YSZ electrode film.

The thickness of the 90 printed layers LSM-YSZ film is estimated to be 9 μm, with its bulk and surface appearing to be very porous, in accordance to the cross-sectional (Figure 4) and top view observations of Figure 5. This is highly encouraging since electrode layers on SOFCs should be porous in order to facilitate gas diffusion through the electrode to the TPB, in contrast to the electrolyte disk that, as presented from top-view SEM, should be dense and impermeable by the SOFC operating gases. It should also be noted that inkjet-printed LSM-YSZ films exhibit great adhesion on the GDC interlayers and substrate, as observed in the friction test (Figure S3 and Video S1), while no delamination or cracking was observed after thermal sintering of the inkjet-printed films, making this report the first to achieve thermally resilient deposition of a water-based inkjet printable LSM-YSZ ink on ceramic substrates. Similar morphology and macroscopic characteristics were observed for the electrodes fabricated by screen printing (SP) of the terpineol-based LSM-YSZ paste, with a difference in the relatively lower precision of the electrode thickness deposition (Figure S4).

## 6 Electrochemical Evaluation of IJP and SP LSM-YSZ|GDC|YSZ Symmetrical Cells

**Figure 6:**
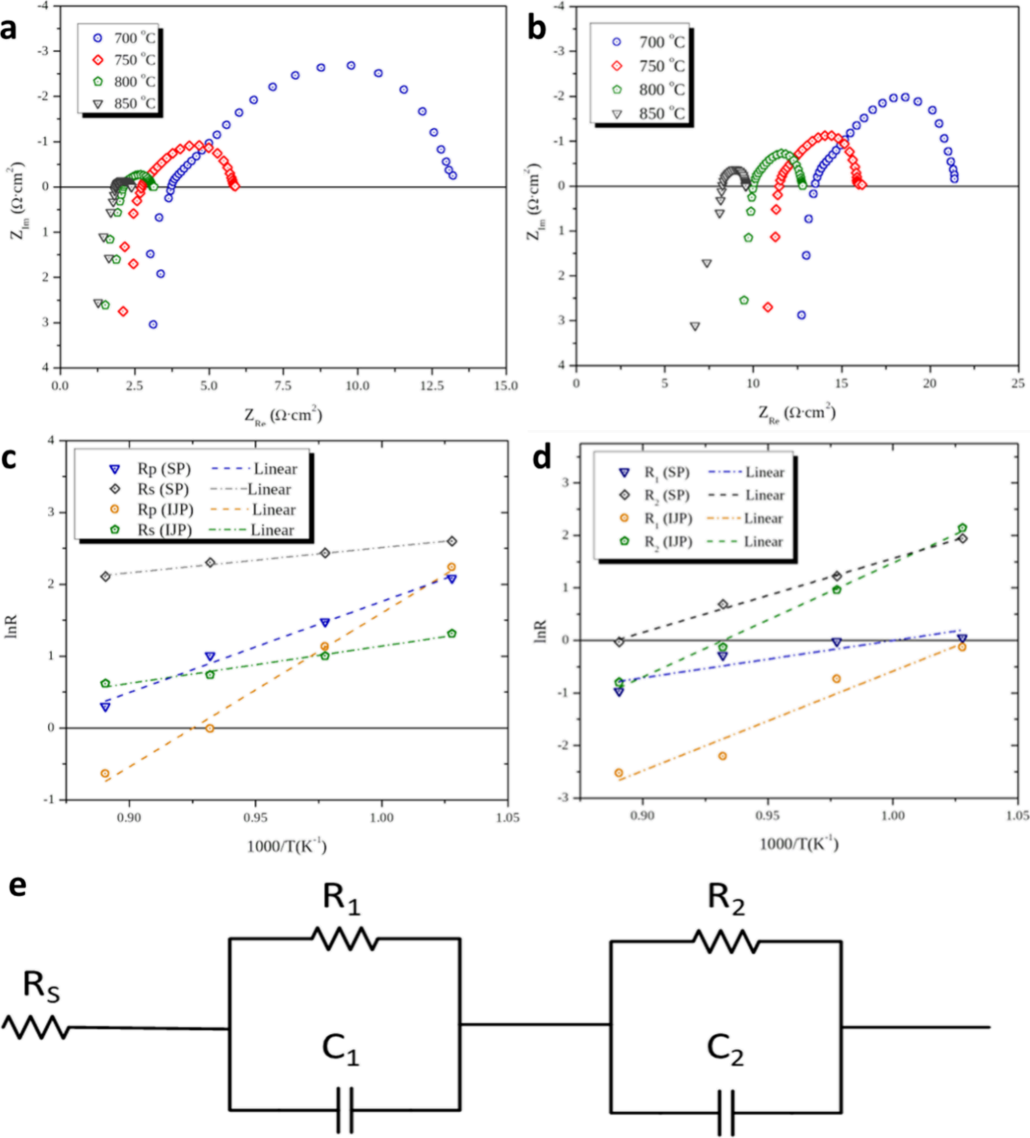
AC impedance spectra of symmetrical solid oxide cells with LSM-YSZ electrodes prepared by (a) IJP and (b) SP. (c) Arrhenius plots of cell resistances are presented in the bottom panels, ohmic and polarization resistances, and (d) electrode polarization components resistances; temperature range: 700–850 °C, gas feed: 150 sccm synthetic air. (e) Equivalent circuit used to fit the impedance spectra. *R*_S_ corresponds to the ohmic resistance of the cell. *R*_1_ and *R*_2_ correspond to the charge transfer (first arc) and mass transfer (second) derived resistances, with their respective constant phase elements modeling the formation of relevant double layers.^40^

The electrochemical performance of the inkjet-printed (IJP) and screen-printed (SP) LSM-YSZ symmetrical cells was evaluated, employing AC impedance spectroscopy and linear scan voltammetry studies at the temperature range of 700–850 °C, under a constant flow of synthetic air (150 cm^3^/min), using a custom-made single chamber cell reactor (see Figure S5). The impedance spectra for both cells at different operation temperatures are presented in Figure 6a (IJP) and Figure 6b (SP), where in all cases, two arcs can be distinguished. At high frequencies, the first arc presents pseudocapacitance values, *C*_1_, in the order of 10^–4^–10^–5^ F cm^–2^, which are associated with charge transfer processes (e.g., O_2_ + 4e^–^ ↔ 2O^2–^).^39−41^ On the other hand, the feature at low frequencies, which is much larger than the first arc shows pseudocapacitance values, *C*_2_, in the order of 10^–3^ F cm^–2^, and is attributed mainly to charged or neutral species mass transfer limitations.^41,42^

In terms of overall cell resistance (*R*_tot_), the IJP cells exhibit clearly lower values than the SP ones at all examined temperatures. This difference is even more pronounced for the ohmic component of the resistance (the initial intercept with the *x*-axis, *R*_S_), where the highest value measured for the IJP cell (3.72 Ω cm^2^ at 700 °C) was substantially lower than the lowest value for the SP sample (7.42 Ω cm^2^), achieved at 850 °C. On the other hand, smaller differences were observed in the electrode polarization resistance, *R*_P_ = *R*_1_ + *R*_2_, where for both *R*_1_ and *R*_2_ components, the resistance values decrease with temperature. Again, in this case, the IJP cell outperformed the SP cell. Since both symmetrical IJP and SP cells were fabricated using identical starting materials, these discrepancies can be ascribed to the differences in the microstructure of electrodes, where the decrease of deposited features in the case of IJP (of micrometer-scale for the drop-by-drop deposition of IJP, versus the millimeter-scale for the layer-by-layer deposition of SP) results in the increased homogeneity of IJP electrodes and electrode/electrolyte interfaces. Detailed values of resistances as well as pseudocapacitance values are available in Table 2.

**Table 2:** Derived Results from Fitting Nyquist Plots with the Equivalent Circuit of Figure 6e^a^

		***R***_**S**_	***R***_**1**_	***R***_**2**_	***R***_**P**_**(*R***_**1**_ **+** ***R***_**2**_**)**	*R*_**tot**_**(*R***_**S**_ **+** ***R***_**p**_**)**	***C***_**1**_	***C***_**2**_
deposition method and cell operation temperature (°C)		Ω·cm^2^	Ω·cm^2^	Ω·cm^2^	Ω·cm^2^	Ω·cm^2^	F·cm^–2^	F·cm^–2^
IJP	700	3.72	0.88	8.50	9.38	13.1	7.20 × 10^–5^	4.71 × 10^–3^
	750	2.72	0.48	2.62	3.1	5.82	13.2 × 10^–5^	2.42 × 10^–3^
	800	2.09	0.11	0.88	0.99	3.08	14.5 × 10^–5^	1.14 × 10^–3^
	850	1.85	0.08	0.45	0.53	2.38	24.6 × 10^–5^	0.55 × 10^–3^
*E*_a_,kJ/mol		42.20	154.29	176.71	174.09	103.06		
SP	700	12.15	0.93	6.16	7.09	19.24	6.63 × 10^–5^	2.50 × 10^–3^
	750	10.30	0.90	3.14	4.04	14.34	10.1 × 10^–5^	1.96 × 10^–3^
	800	9.03	0.68	1.79	2.47	11.50	1.07 × 10^–5^	0.86 × 10^–3^
	850	7.42	0.34	0.87	1.21	8.63	11.38 × 10^–5^	0.44 × 10^–3^
*E*_a_,kJ/mol		28.53	57.84	114.62	103.12	46.76		

a Ohmic resistances (*R*_S_), polarization resistances (*R*_P_) and its components (*R*_1_ and *R*_2_), overall cell resistances (*R*_tot_) along with the corresponding activation energies (*E*_a_), and pseudo-capacitance (*C*_1_ and *C*_2_) values, for inkjet-printed (IJP) and screen-printed (SP) symmetrical LSM-YSZ|GDC|YSZ cells at different operation temperatures (700–850 °C).

A study published in 2020 by Pesce et al.^43^ reported on a similar symmetric single cell, with conventional deposition of the commercially available LSM-YSZ on a novel YSZ electrolyte fabricated by the stereolithography (SLA) printing method, with electrolyte thickness (270 μm) close to the currently presented work. Results from the temperature range of 700–850 °C under synthetic air flow indicate a similar Nyquist plot comprised of two arcs, with values of *R*_S_ and *R*_P_ being up to three times higher compared to the current work. These results illustrate that electrodes derived from the currently presented inkjet-printing method for fabrication of the LSM-YSZ air electrodes onto a commercial YSZ electrolyte disk can perform better than LSM-YSZ electrodes, which are conventionally deposited (painted) even in the case where a state-of-the-art SLA-printed electrolyte is utilized.

An analogous study by a different research group fabricated LSCF-GDC symmetric single cells supported on GDC electrolytes of about 600 μm thickness, by infiltration using inkjet printing.^24^ Their Nyquist plots at 550 °C also comprised two arcs, a smaller one at high frequencies and a larger one at lower frequencies, with values of *R*_S_ and *R*_P_ similar to those obtained in this work at 700 °C.

In the graphs presented in Figure 6c,d, the natural logarithm of the individual resistances is plotted versus the inverse absolute temperature (i.e., Arrhenius plots). All resistance values decrease with increasing temperature, but the activation energy (linear slope) of each is different, with the lowest activation energy observed for the ohmic resistance (*R*_S_) and the highest for *R*_2_ (also reflected in *R*_P_). In general, the activation energies calculated for the IJP cells were higher than those of the SP ones; the greatest difference being observed for *R*_1_, attributed to charge transfer polarization, and the smallest for *R*_S_, the ohmic resistance. These activation energies are roughly an order of magnitude higher than those reported for LSCF-GDC symmetrical cells.^24^

A more detailed comparison of this work with the literature is presented in Table 3.

**Table 3:** Comparison Table on Electrochemical Performance and Ink Types between This Work and Previously Reported Literature on Inkjet Printed SOFC Cathodes^a^

**electrolyte (thickness)**	**support material**	**electrode (thickness)**	**ink type**	***R***_**S**_**(Ω cm**^**2**^**)**	***T* (°C)**	**ref**
YSZ (16 um)	NiO-YSZ	LSM-YSZ (4 um)	S-Org	0.2	800	(27)
YSZ (16 um)	NiO-YSZ	LSM-YSZ (10 um)	S-Org	0.4	850	(28)
GDC (50 um)	NiO-GDC	LSCF-GDC (3 um)	S–H_2_O	n/a	600	(23)
YSZ/SDC (n/a)	NiO-YSZ	SSC-SDC (10 um)	S–H_2_O	0.25	750	(32)
YSZ/GDC (10 um)	NiO-YSZ	LSCF (5 um)	S–H_2_O	0.26	600	(44)
YSZ/GDC (10 um)	NiO-YSZ	LSCF-GDC (14 um)	S-Eth	0.15	650	(31)
YSZ (10 um)	NiO-YSZ	LSM-YSZ (150 um)	S-Org	0.09	788	(20)
GDC (600 um)	symmetric	LSCF-GDC (15 um)	S-Org	1.27	550	(24)
YSZ/GDC (242 um)	symmetric	LSM-YSZ (9 um)	S–H_2_O	1.85	850	our work

a Ink type: S = suspension ink; Solvent: H_2_O = water; Org = organic; Eth = ethanol.

**Figure 7:**
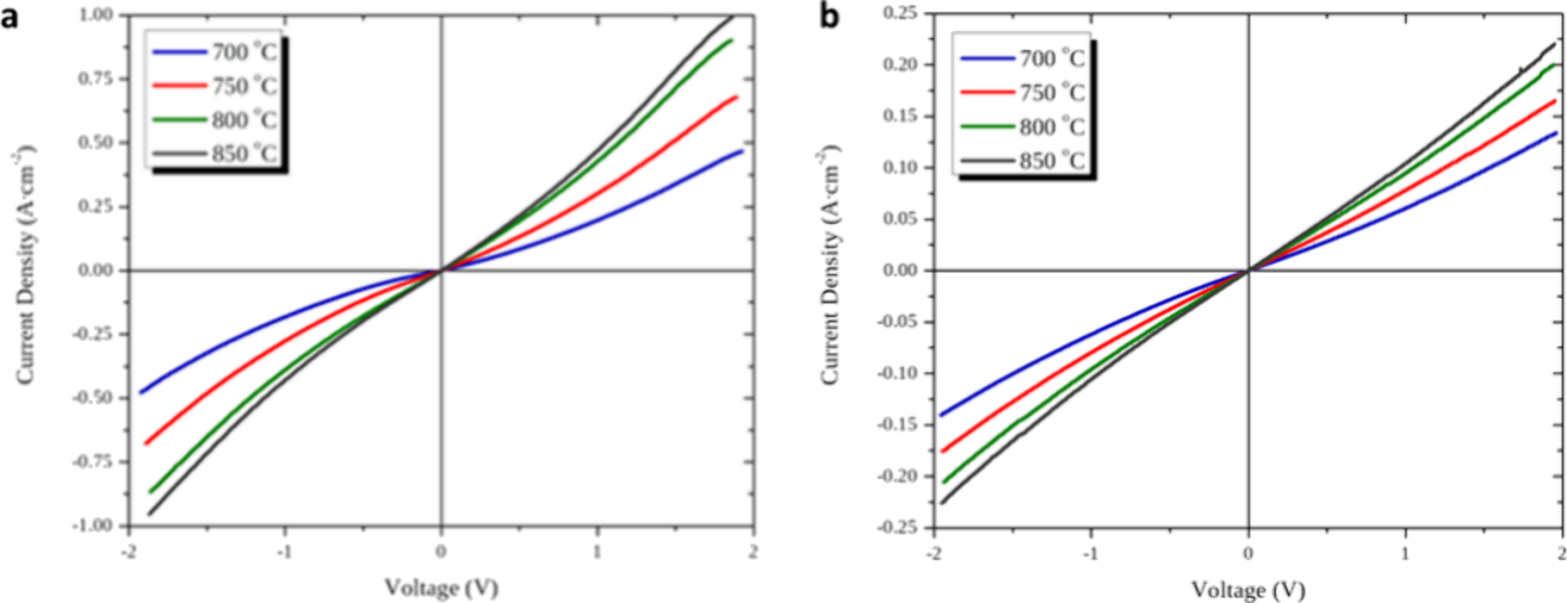
Current–voltage plots of symmetrical cells prepared by (a) IJP and (b) SP; temperature: 700–850 °C, gas feed: 150 sccm synthetic air.

The above-discussed observations for IJP- and SP-deposited LSM-YSZ electrodes are reflected in the obtained cell performances displayed in Figure 7. Here, the achieved current density is plotted against the applied scanning voltage (from −2 to 2 V) for cells with IJP (Figure 7a) and SP (Figure 7b) electrodes. The current density peaks obtained with the IJP cell are much higher than those with the SP cell at each temperature examined. Indicatively, at 700 °C the highest current density obtained with the SP cell was 140 mA·cm^–2^ at 2 V, as opposed to 466 mA cm^–2^ for the IJP cell. All the *I*–*V* curves are smooth and symmetrical for the negative and positive polarization operation, following the pattern of the Butler–Volmer equation with an activation overpotential of about 0.3 V, which corresponds to the oxygen reduction charge transfer reaction occurring at the three-phase-boundary sites.

## 7 Conclusions

In the present study, high-performance and high-quality LSM-YSZ thin films were developed by inkjet printing and tested as oxygen electrodes for SOFCs in symmetrical LSM-YSZ|GDC|YSZ single cells. LSM-YSZ nanoparticles (with an average size of 170 nm) were produced from commercial powder via ball milling, while the physicochemical properties of the produced water-based LSM-YSZ nanoparticle ink were systematically examined to assess its stability and printability The formulated water-based LSM-YSZ nanoparticle ink exhibited high stability (up to 60 days of storage) without any measurable aggregation of particles and fully reversible precipitation. Deposition parameters of the print-head controlling the ink deposition such as operating temperature, jetting voltage, and the jetting waveform were optimized to achieve continuous generation and uniformity of droplets toward stable inkjet printing. The use of a single jetting nozzle was established to optimize the printing process, resulting in depositions with better accuracy, at a drop spacing of 31 μm and 819 dpi resolution. Finally, LSM-YSZ electrodes deposited by inkjet printing on dense YSZ substrates using GDC as buffer interlayer exhibited superior performance for the oxygen reduction reaction in all temperatures examined (700–850 °C) compared to the screen-printed cell. The obtained current densities in the IJP cell were almost five times higher, up to 1 A/cm^2^ at 2 V cell potential and 850 °C, compared to the SP cell reflecting the observed differences in overall, ohmic, and electrode polarization resistances. The present study showcases the potential of inkjet printing technique for the deposition of LSM-YSZ thin films for enhanced oxygen electrodes in SOFCs.

## Supporting Information Available

Friction test of an inkjet-printed LSM-YSZ film (MP4)
Energy-dispersive X-ray spectroscopy and elemental composition of the LSM-YSZ powders before and after ball milling; SEM images of printed lines at different drop spacing values; screenshots from the film adhesion test video where the adhesion of the inkjet-printed LSM-YSZ films after the thermal treatment is exhibited; cross-sectional SEM image of screen-printed LSM-YSZ; schematic representation of the homemade single chamber cell reactor (PDF)
