# Supplementary material for: Inkjet-Printed LSM-YSZ Thin Films for Enhanced Oxygen Electrodes in Solid Oxide Fuel Cells
Source: Energy Fuels. 2024 Jul 12;38(15):14621–31. doi: 10.1021/acs.energyfuels.4c00673 (PMC11299173; doi:10.1021/acs.energyfuels.4c00673)
Supplement: Supplementary file 2 — ef4c00673_si_002.pdf [file ef4c00673_si_002.pdf]

Supporting Information

Inkjet-Printed LSM-YSZ thin films for enhanced oxygen electrodes in SOFCs

*Michalis Charalampakis[a,b], Leila Zouridi[b,c], Ioannis Garagounis[d], Anastasios Vourros[d], George E. Marnellos[d,e], Vassilios Binas[b,f]**

a Department of Chemistry, University of Crete, Vasilika Vouton, 70013 Heraklion Greece

b Institute of Electronic Structure and Laser, Foundation for Research and Technology-Hellas, N. Plastira 100, Vasilika Vouton, 70013 Heraklion Crete, Greece

c Department of Materials Science and Technology, University of Crete, Vasilika Vouton, 70013 Heraklion, Greece

d Chemical Process & Energy Resources Institute, Centre for Research & Technology Hellas, 6th km Harilaou-Thermis, 57001, Thessaloniki, Greece

e Department of Chemical Engineering, Aristotle University of Thessaloniki, University Campus, 54124 Thessaloniki, Greece

f Physical Chemistry Laboratory, Chemistry Department, Aristotle University of Thessaloniki, Faculty of Sciences, 54124, Thessaloniki, Greece

KEYWORDS: inkjet printing, fuel cells, LSM-YSZ, SOFC, ink development, water-based ink, symmetrical cathode cells, oxygen electrodes, water-based ink

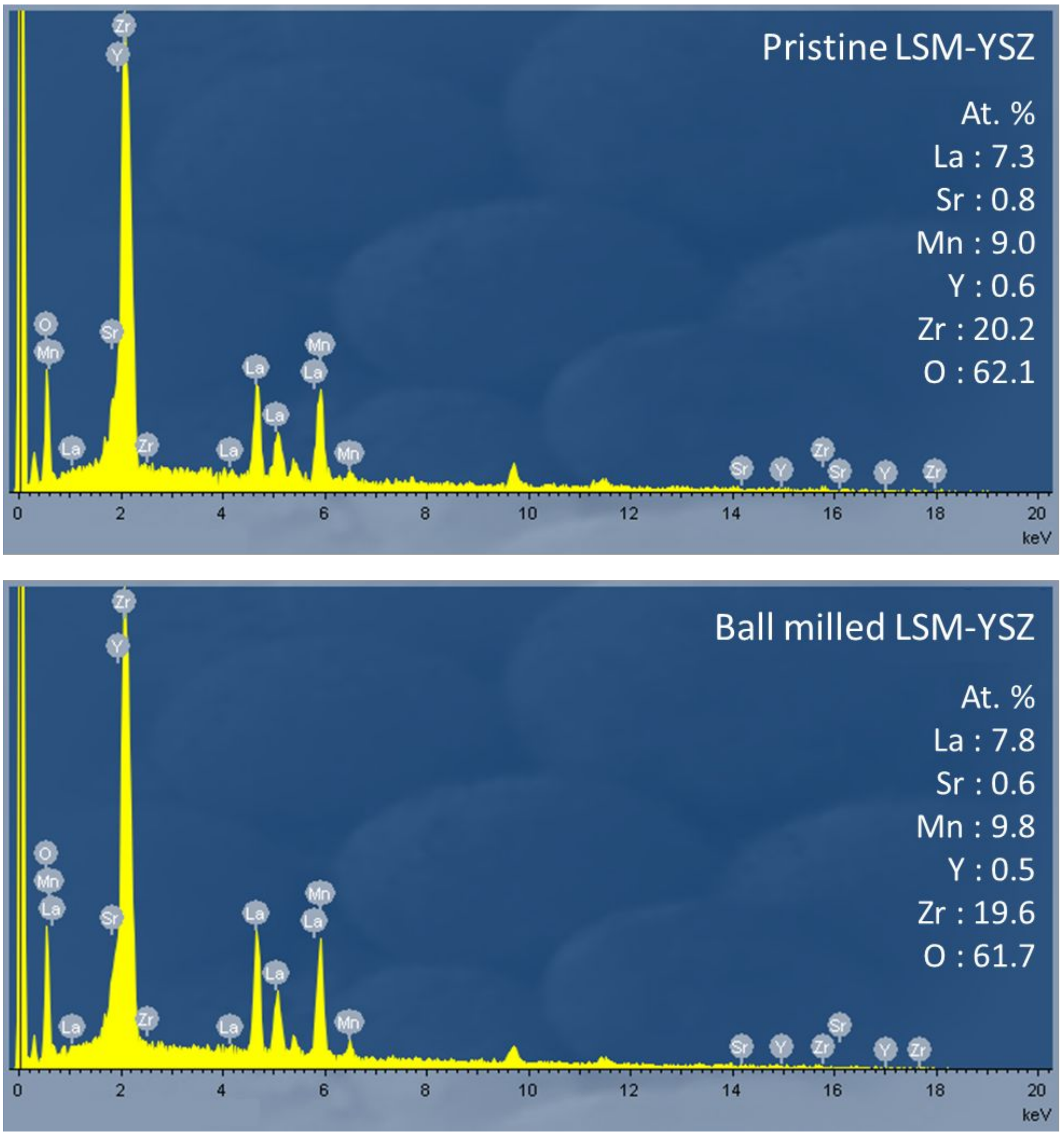

Figure S1: Energy-Dispersive X-Ray spectroscopy and elemental composition of the LSM-YSZ powders before and after ball milling.

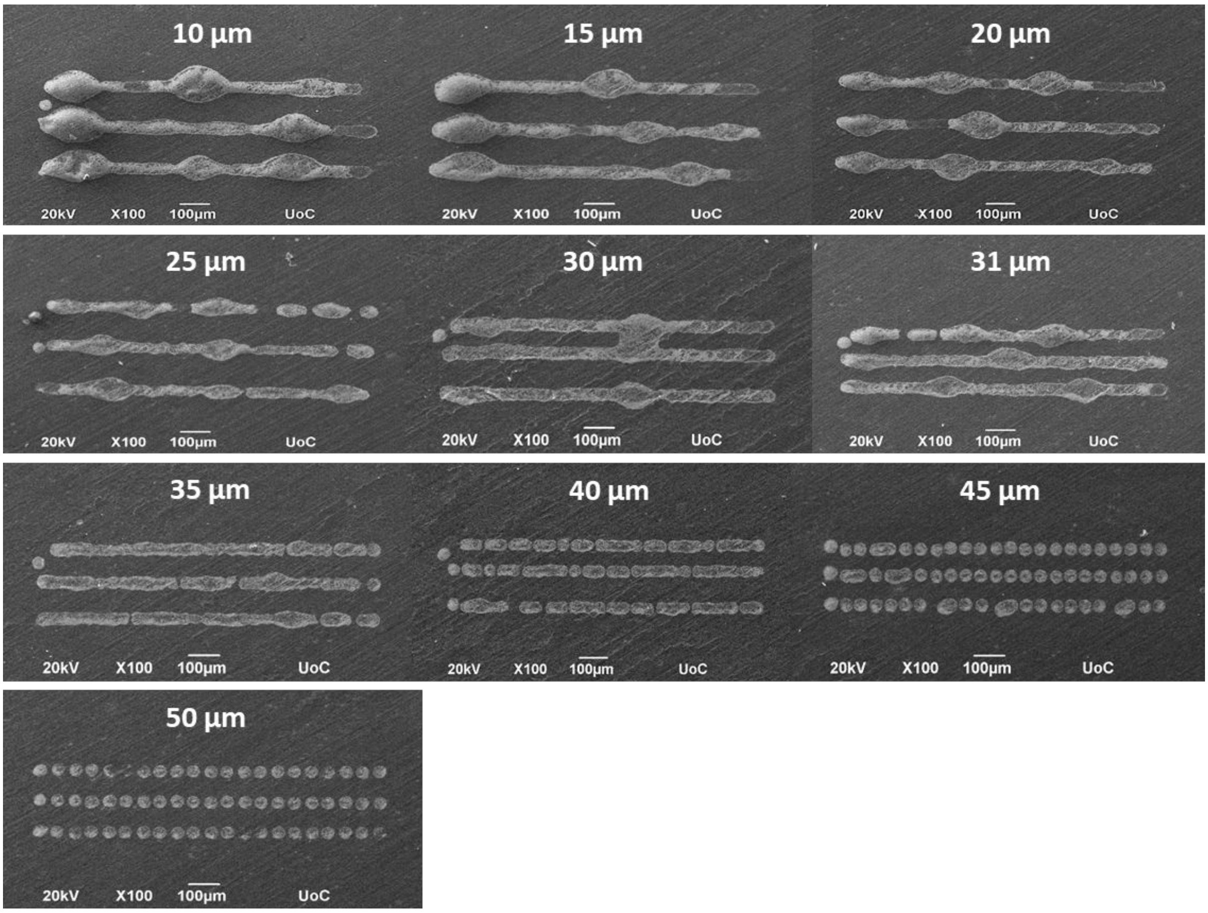

Figure S2: SEM images of inkjet-printed lines at different drop spacing values.

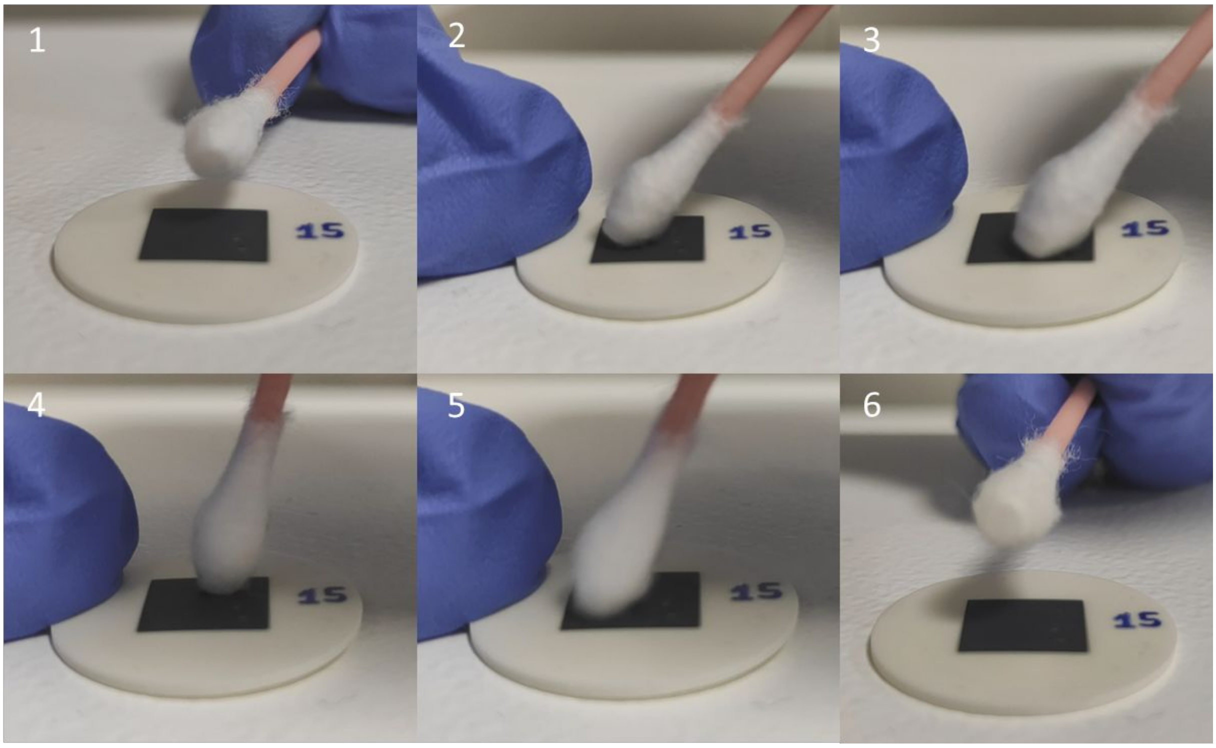

Figure S3: Screenshots from the film adhesion test video where the adhesion of the inkjet-printed LSM-YSZ films after the thermal treatment is exhibited.

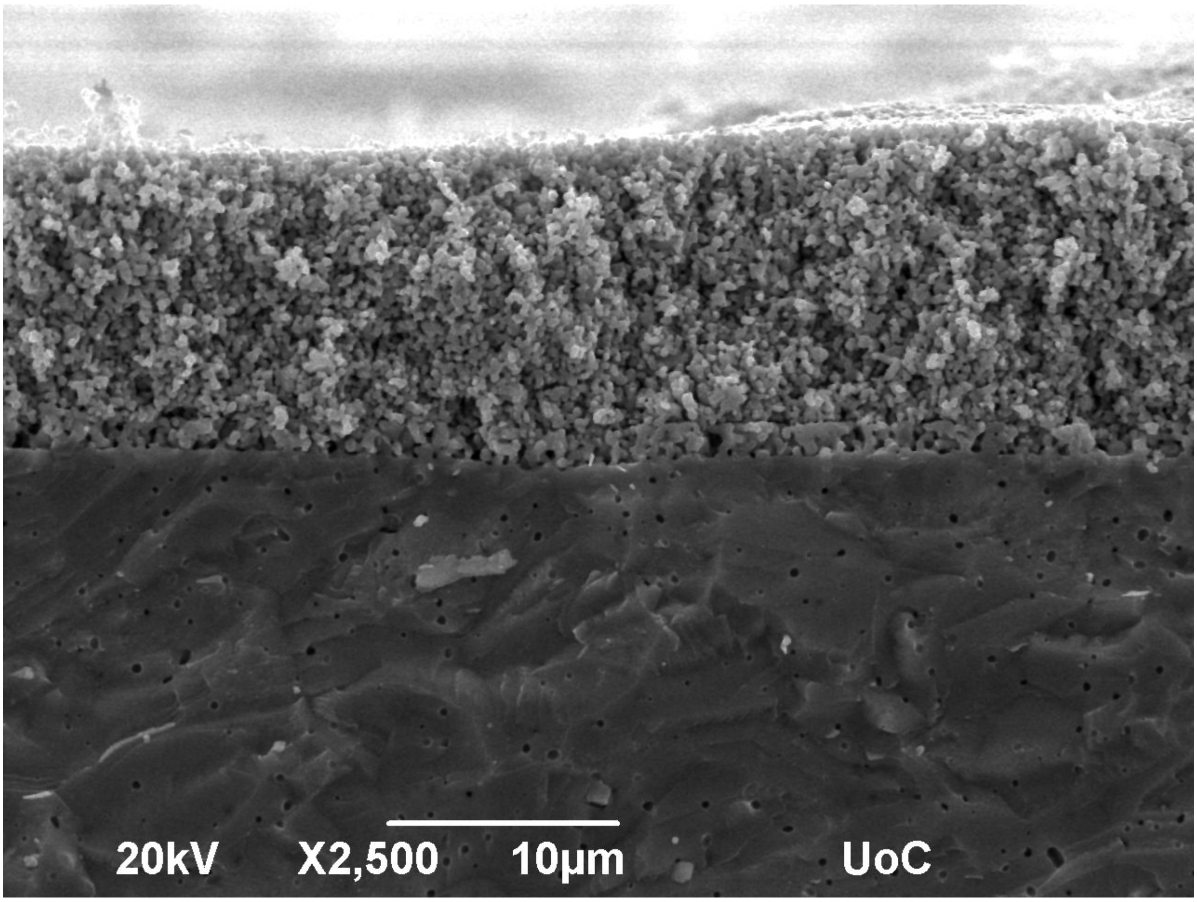

Figure S4. Cross-section SEM image of screen-printed LSM-YSZ.

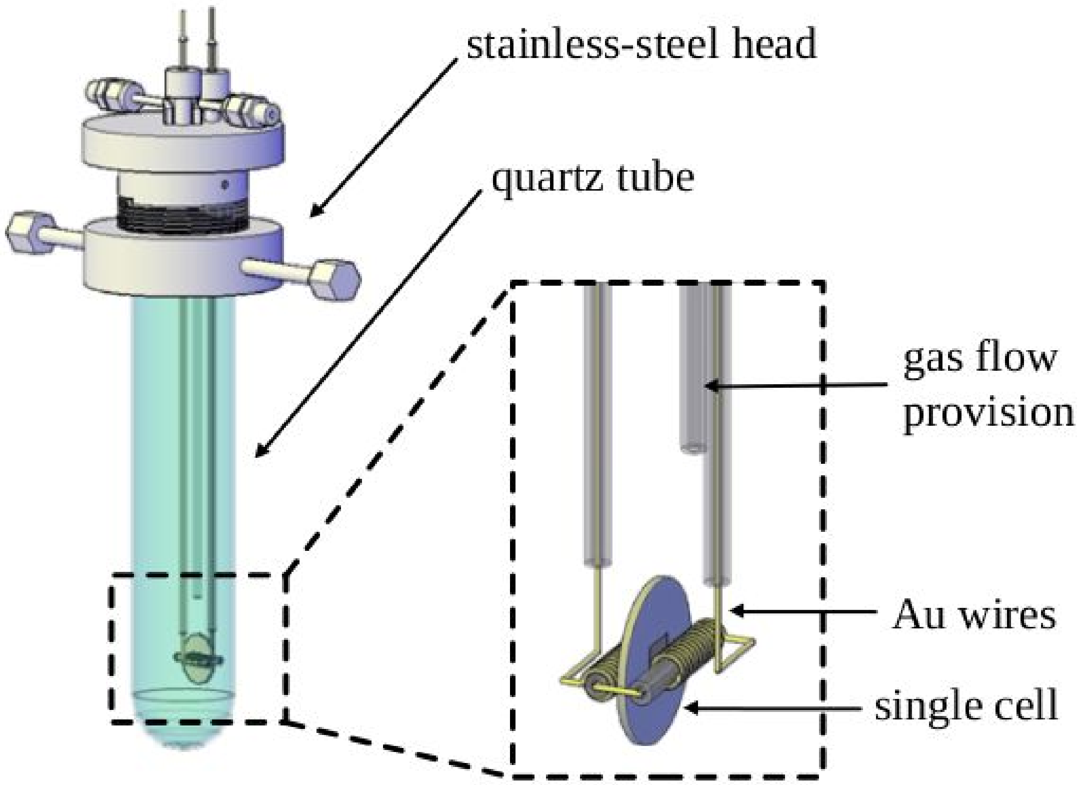

Figure S5. Schematic representation of the homemade single chamber cell reactor.
